# *Euphorbia kansui* Attenuates Insulin Resistance in Obese Human Subjects and High-Fat Diet-Induced Obese Mice

**DOI:** 10.1155/2017/9058956

**Published:** 2017-10-04

**Authors:** Seung-Wook Lee, Hyun-Young Na, Mi Hyeon Seol, Mia Kim, Byung-Cheol Lee

**Affiliations:** ^1^Department of Clinical Korean Medicine, Graduate School, Kyung Hee University, 26 Kyungheedae-ro, Dongdaemun-gu, Seoul 02447, Republic of Korea; ^2^Department of Cardiovascular and Neurologic Disease (Stroke Center), College of Korean Medicine, Kyung Hee University, 23 Kyungheedae-ro, Dongdaemun-gu, Seoul 02447, Republic of Korea

## Abstract

**Background:**

Obesity is a main cause of insulin resistance (IR), metabolic syndrome, and fatty liver diseases. This study evaluated* Euphorbia kansui* radix (*Euphorbia*) as a potential treatment option for obesity and obesity-induced IR in obese human and high-fat diet- (HFD-) induced obese mice.

**Methods:**

In the human study, we analyzed the body weight change of 14 patients who took a single dose of 6 g of* Euphorbia* powder. In the animal study, male mice were divided into three groups: normal chow, HFD, and* Euphorbia* (high-fat diet and 100 mg/Kg* Euphorbia* once per week). Body weight, epididymal fat pad weight, fasting blood glucose, fasting insulin, HOMA-IR, and oral glucose tolerance test were measured. Also, macrophage infiltration and expression of CD68, tumor necrosis factor- (TNF-) *α*, interferon- (IFN-) *γ*, and interleukin- (IL-) 6 genes in the liver and adipose tissue were analyzed.

**Results:**

The human study showed that* Euphorbia* has a potential effect on body weight loss. In the in vivo study, body weight, epididymal fat weight, glucose level, IR, expression of CD68, TNF-*α*, IFN-r, and IL-6 genes, and macrophages in liver and adipose tissue were significantly reduced by* Euphorbia*.

**Conclusions:**

These results suggest that* Euphorbia* attenuates obesity and insulin resistance via anti-inflammatory effects.

## 1. Introduction

Obesity is rapidly increasing worldwide [1]. Increased visceral adipose tissue confers a proinflammatory milieu and insulin resistance (IR) [2], which is directly or indirectly related to various health problems including type 2 diabetes mellitus (T2DM), hypertension, dyslipidemia, and fatty liver diseases, resulting in an annual cost of about 150 billion dollars [3].

Many efforts, including lifestyle changes and antiobesity drugs, have been applied for obesity treatment. However, lifestyle change is not ensured to maintain weight loss for more than a few years, and the use of drugs has been limited by side effects such as negative mood changes, suicidal thoughts, and gastrointestinal or cardiovascular complications [4, 5]. Therefore, new efforts, including herbal medicines, can provide an alternative therapy for this medical challenge.

The root of* Euphorbia kansui *Liou* (Euphorbia)* is an herb that has been commonly used in China for thousands of years. It is in the cathartic (resolving water retention through stool) category of the Chinese medical literature [6]. In clinics, it is often used to treat ascites caused by hepatocirrhosis [7], pancreatitis [8, 9], and intestinal obstruction [10]. In recent years, there have been reports of its antitumor [11] and antiviral role [12, 13] as well as its ability to regulate the immune system [14]. Moreover, recent studies have revealed that* Euphorbia* has anti-inflammatory [15], antidiabetic [16], and antiobesity effects [17].

In this study, we evaluated the effects of* Euphorbia* on obesity, glycemic control, and IR. In addition, we addressed the safety issue of* Euphorbia *and investigated its mechanisms of action, especially with respect to the inflammatory reaction and macrophage infiltration in liver and adipose tissue.

## 2. Materials and Methods

### 2.1. Preparation of* Euphorbia*

*Euphorbia* was purchased from the Department of Pharmaceutical Preparation of Kyung Hee University Oriental Medical Hospital (Seoul, South Korea). The original source of the* Euphorbia* was Kyung Hee Herb Pharm (WonJu, South Korea). Drug quality was tested based on the standards of the Korea Food and Drug Administration and the standards of the hospital. Extractions were prepared from dried* Euphorbia*.* Euphorbia* (100 g) was added to 1,500 ml 80% ethanol and boiled for 2 hours using a heating mantle. The sieve-filtered solution was filtered into a 500 ml flask and concentrated with a rotary evaporator (model NE-1, EYELA Co., Tokyo, Japan). The solution was freeze-dried, and the* Euphorbia* extract was stored at room temperature. The final collection yield was 9.6%.

### 2.2. Human Study

The inclusion criteria were as follows: BMI over 23, age between 19 and 65 years, ability to provide informed consent, and stable health according to the opinion of the investigator. The exclusion criteria included pregnant or lactating women, participants who had undergone another obesity treatment in the previous 3 months, participants of clinical trials within the previous 3 months, clinically significant new illness in the 1 month before screening, or not being suitable for participating in the study in the opinion of the investigator including an existing physical or mental condition, significant change in smoking habits within the previous 3 months, and diseases including epilepsy, uncontrolled hypertension, or hypotension, cerebrovascular diseases, and clinically significant abnormal hepatic (e.g., AST or ALT greater than 2.5 × ULN or total bilirubin greater than 1.5 × ULN) or renal function lab tests (e.g., creatinine greater than 1.25 × ULN), suggestive of hepatic or renal impairment.

Eligible patients recruited from January 2014 to October 2014 were given a single 6 g dose of raw* Euphorbia* powder with simple dietary consultations at the Korean Medicine Hospital of Kyung Hee University, Seoul, South Korea. The consultations include taking 3 meals a day regularly and prohibiting confections. Patients were analyzed for body weight (BW), height, waist-hip ratio, body fat percentage, aspartic aminotransferase (AST), alanine aminotransferase (ALT), gamma glutamyltranspeptidase (GGT), blood urea nitrogen (BUN), and creatinine before and one month after treatment. Body mass index (BMI) and CockCroft-Gault estimated glomerular filtration rate (eGFR) were calculated from the above data. Furthermore, other side effects were noted after* Euphorbia* administration. BW, BMI, waist-hip ratio, and body fat percentage of patients were estimated using a body composition analyzer (Inbody Co., Seoul, Korea). This study was approved by the International Review Board of Kyung Hee Hospital (KOMCIRB-2014-06).

### 2.3. Animals and Diets

The animals used in this study were 19–21 g, 5-week-old, male C57BL/6 mice (Central LabAnimals, Inc., Korea). To determine the optimal dose of* Euphorbia*, thirty-five mice fed normal chow (NC) with 10% fat were randomized to be treated with the following dose levels of* Euphorbia* with five mice per dose level: no treatment, 20 mg/kg, 50 mg/kg, 100 mg/kg, 200 mg/kg, 500 mg/kg, and 1000 mg/kg of* Euphorbia* were orally administered once weekly for 28 days. Upon completion of the study, all mice were weighed and sacrificed, and the liver, spleen, and kidney were harvested and weighed. After 28 days of treatment, an 18.2% reduction of body weight was achieved with the 100 mg/kg dose level compared to the no treatment control group. No differences of liver, spleen, and kidney weight were observed with 100 mg/kg dose* Euphorbia*, finally, the 100 mg/Kg of* Euphorbia* was selected for main experiment. With free access to water and food, the mice were fed a high-fat diet (HFD) with 60% fat for 13 weeks in order to induce obesity, except in the group NC with 10% fat. After 8 weeks of the HFD diet, the HFD groups showed significant body weight differences compared to the NC-fed group (28.98 ± 1.40 g and 41.26 ± 1.14 g, resp., *p* < 0.001). The HFD-fed mice were randomly assigned to two groups: HFD and* Euphorbia* groups (*n* = 5 in each). For the remaining 5 weeks of the experiment, the* Euphorbia* group was fed* Euphorbia* (100 mg/kg) extract once a week, while the NC and HFD groups were administered normal saline. All experiments were carried out in accordance with guidelines of the Korean National Institute of Health Animal Facility. The Animal Care Committee at Kyung Hee University approved all protocols used in this study (KHMC-IACUC 14-025).

### 2.4. Body and Epididymal Weight Measurement

Body weight (BW) was recorded at the beginning and end of the experiment using an electronic scale (CAS 2.5D, Seoul, Korea) and was measured at the same time in the morning every day. The mice were killed at 13 weeks after blood collection by heart puncture. The epididymides and livers were removed rapidly and weighed.

### 2.5. Insulin Concentration and Insulin Resistance Measurement

At the 11th week of the experimental period, we withdrew blood from the tail vein of mice after 6 hours of fasting. Serum insulin concentration was measured using an ultrasensitive mouse insulin ELISA kit (Crystal Chem Inc., USA). Insulin resistance was calculated using the following equation: HOMA-IR = fasting blood glucose (mg/dl) × fasting blood insulin (mg/ml) × 0.0717225161669606.

### 2.6. Oral Glucose Tolerance Test (OGTT)

At the 12th week, an oral glucose tolerance test was performed after the mice had undergone 14 hours of fasting. The fasting (baseline) blood glucose measurement was performed by applying a drop of tail blood to a strip-operated blood glucose sensor (ACCU-CHECK Performa, Australia). Glucose (2 g/kg body weight) was administered orally to each animal. Tail vein blood samples were withdrawn at 30, 60, and 120 minutes after glucose administration.

### 2.7. Stromal Vascular Cell (SVC) Segregation

Epididymal fat pad tissue, obtained at week 13 of the experimental period, was mixed with a solution of phosphate-buffered saline (PBS, Gibco, USA) and 2% bovine serum albumin (BSA, Gibco, USA). After cutting the fat pad into 1~2 mm sized round shapes with scissors, collagenase (Sigma, USA) and DNase I (Roche, USA) were added. A 100 *μ*m cell strainer (BD Biosciences, USA) was used to remove extraneous tissue.

### 2.8. Fluorescence Activated Cell Sorting (FACS) Analysis of Adipose Tissue Macrophages (ATMs)

The number of SVCs obtained from the adipose tissue was counted by cellometer (Nexcelom Bioscience LLC, USA), and every sample was set at a concentration of 10^6^ cells. FcBlock (BD Pharmingen, USA) was mixed with the sample at a ratio of 1 : 100, and the reaction was performed for 10 minutes. Fluorophore-conjugated antibodies were added to the shaded state and reacted for 20 minutes. The following antibodies were used: CD45-APC Cy7 (Biolegend, USA), CD68-APC (Biolegend, USA), CD11c-phycoerythrin (CD11b-PE, Biolegend, USA), and CD206-FITC (Biolegend, USA). The samples were analyzed by FACSCalibur (BD Biosciences, USA). A FlowJo (Tree Star, Inc., USA) was used to analyze the percentage of macrophages.

### 2.9. Immunofluorescence Staining of Stromal Vascular Cells

SVCs were plated onto glass coverslips for 2 hours, and nonadherent cells were removed by washing in PBS. The cells were fixed in 10% formalin for 20 minutes and then blocked in 2% BSA in PBS. Lipid staining was performed by incubation of fixed cells in 1 ug/ml Bodipy (Sigma-Aldrich) in PBS for 5 minutes. Coverslips were mounted on slides with Vectashield (Vector Laboratories, Burlingame, CA) and imaged under fluorescence microscopy (Olympus BX51, Olympus, Japan).

### 2.10. Immunohistochemistry

Immunohistochemistry was performed on 10% buffered formalin-fixed, paraffin-embedded liver tissues using antibodies against F4/80 (1 : 100, Genetex, Irvine, CA), followed by incubating in biotinylated anti-mouse/rabbit/goat IgG (H + L), made in horse (Vector Laboratories, Inc.). Liver tissue sections were consecutively stained with avidin-biotin horseradish peroxidase complex (Vectastain ABC ELITE kit; Vector Laboratories, Burlingame, CA) for 15 min before a substrate solution of 3,3′-diaminobenzidine tetrahydrochloride (DAB; Sigma Chemical, St. Louis, MO) was added. The sections were photographed under an Olympus photomicroscope (Olympus BX-50, Olympus Optical, Tokyo, Japan). The percentage of F4/80-positive area in liver tissues was analyzed using the ImageJ software (NIH, USA).

### 2.11. RNA Extraction and Real-Time Reverse Transcriptase-Polymerase Chain Reaction

Mini RNA Isolation IITM (ZYMO RESEARCH, CA, USA) was used for separation of RNA from liver and epididymal fat pads. To evaluate the gene expression of CD68 (macrophage marker), tumor necrosis factor-*α* (TNF-*α*), interferon-*γ* (IFN-*γ*), and interleukin-6 (IL-6) in adipose tissue, we performed quantitative reverse transcriptase-polymerase chain reaction (qRT-PCR). Primers for the analysis were designed as follows: CD68, 5′-TTCTGCTGTGGAAATGCAAG and 5′-AGAGGGGCTGGTAGGTTGAT; TNF-*α*, 5′-TTCTGTCTACTGAACTTCGGGGTGATCGGTCC and 5′-GTATGAGATAGCAAATCGGCTGACGGTGTGGG; IFN-*γ*, 5′-ACTGGCAAAAGGATGGTGAC and 5′-TGAGCTCATTGAATGCTTGG; IL-6, 5′-AACGATGATGCACTTGCAGA and 5′-GAGCATTGGAAATTGGGGTA; GAPDH, which was used as a housekeeping gene, 5′-AGTCCATGCCATCACTGCCACC and 5′-CCAGTGAGCTTCCCGTTCAGC. The threshold cycle (TC) of each gene expression, determined by SDS Software 2.4 (Applied Biosystems®, USA), was converted into Relative Quantitation (RQ) based on GAPDH, and the calculated fold change value was used for gene expression analysis. The fold change value of the experimental group was converted based on the NC group value, which was considered to be one.

### 2.12. Statistical Analysis

All calculations were performed using GraphPad PRISM 5 (GraphPad Software, Inc., San Diego, CA, USA). All values are expressed as mean ± SE. The significance of differences between groups was determined using one-way analysis of variance (ANOVA), followed by Tuckey's post hoc test. All *p* values were two-tailed, and significance was set at *p* < 0.05. 

## 3. Results

### 3.1. Effects of* Euphorbia* in Overweight or Obese Human Patients

The BW changes of 14 patients were obtained at one month after one-time treatment with 6 g* Euphorbia*. There was significant decrease in BW and BMI after* Euphorbia* powder administration (65.37 ± 3.53 kg versus 64.10 ± 3.49 kg for BW and 24.93 ± 0.65 versus 24.28 ± 0.58 for BMI, *p* < 0.05) (Table 1). There was no significant difference in BUN, creatinine, eGFR, AST, ALT, GGT, protein, albumin, fasting glucose, and HbA1c before and after administration with* Euphorbia* (Table 1). As* Euphorbia* is traditionally classified as a cathartic herb, patients underwent an average of 0.92 ± 0.27 cases of vomiting and 8.08 ± 1.24 cases of diarrhea. There were several mild side effects including 1 report of mild abdominal pain and 6 cases of abdominal discomfort, which diminished after a few days.

### 3.2. Effects of* Euphorbia* on BW and Epididymal Fat Weight Changes in Mice

**Figure 1:**
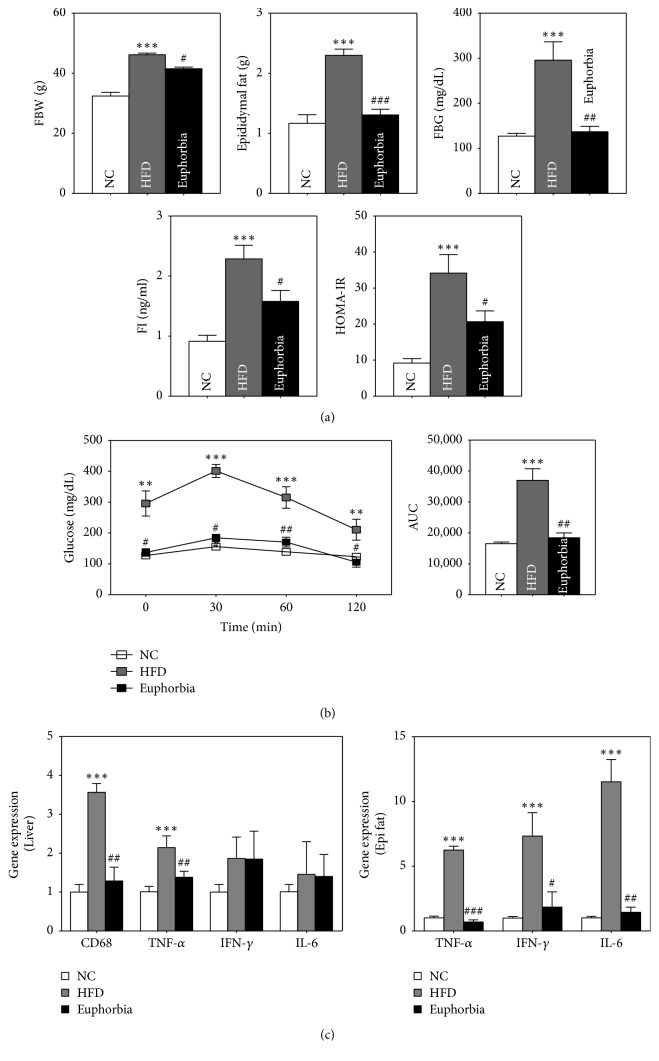
Main results of the* in vivo* study. (a) Fasting body weight (FBW), epididymal fat weight, fasting blood glucose (FBG), fasting insulin (FI), HOMA-IR of normal chow NC, HFD, and* Euphorbia* groups. (b) Oral glucose tolerance test (OGTT) and area under curve (AUC) of the three groups. (c) Expression of CD68, TNF-*α*, IFN-*γ*, and IL-6 genes in the three groups. ^*∗∗*^*p* < 0.01 and ^*∗∗∗*^*p* < 0.001, HFD compared with NC; ^#^*p* < 0.05, ^##^*p* < 0.01, and ^###^*p* < 0.001,* Euphorbia* compared with HFD. NC: NC group; HFD: HFD group;* Euphorbia*:* Euphorbia* group.

The fasting body weight (FBW) of the HFD group increased significantly compared to that of the NC group (*p* < 0.05), and* Euphorbia* group showed a decrease in BW compared to the HFD group (*p* < 0.05) (Figure 1(a)). The weight of epididymal fat also was significantly increased in the HFD group compared to the NC and was decreased by* Euphorbia* (*p* < 0.001) (Figure 1(a)).

### 3.3. Effects on Blood Glucose and Insulin Resistance in Mice

Fasting blood glucose (FBG) was significantly higher in the HFD group than NC group (*p* < 0.001), and it was significantly lower in the* Euphorbia* group than HFD group (*p* < 0.01). Fasting insulin concentration (FI) was significantly higher in the HFD group compared to the NC group (*p* < 0.001) and was significantly lower in the* Euphorbia* group compared to the HFD group (*p* < 0.05). Insulin resistance calculated by HOMA-IR was significantly higher in the HFD group than NC group (*p* < 0.05), while the HOMA-IR of the* Euphorbia* group was significantly lower than that HFD group (*p* < 0.01) (Figure 1(a)).

OGTT, conducted at 12 weeks after initiation of the study, showed the highest blood glucose concentration at 30 minutes in every group and decreased gradually. The 30-, 60-, and 120-minute glucose levels of the HFD group were elevated significantly compared to those of the NC group (*p* < 0.05) and were significantly lower in the* Euphorbia* group than in the HFD group (*p* < 0.05). The area under the curve (AUC) also showed similar tendencies, with the AUC of the HFD group being higher than that of the NC group, while that of the* Euphorbia* group was significantly lower than that of the HFD group (*p* < 0.01) (Figure 1(b)).

### 3.4. Effects on Expression of CD68, TNF-*α*, IFN-*γ*, and IL-6 Genes

The gene expression of CD68 in liver was significantly higher in the HFD group than NC group (*p* < 0.001) and was significantly lower in the* Euphorbia* group compared to the HFD group (*p* < 0.01). And TNF-*α* in liver and epididymal fat was significantly higher in the HFD group than NC group (*p* < 0.001), and* Euphorbia* significantly lowers TNF-*α* gene expression compared to the HFD group (*p* < 0.001). IL-6 expression in epididymal fat was also higher in the HFD group than in the NC group and decreased significantly in the* Euphorbia* group compared to the HFD group (*p* < 0.05). The IFN-*γ* in epididymal fat expression was significantly increased in the HFD group compared to the NC and* Euphorbia* groups (*p* < 0.05) (Figure 1(c)).

### 3.5. Effects on Macrophages in Liver and Adipose Tissue

**Figure 2:**
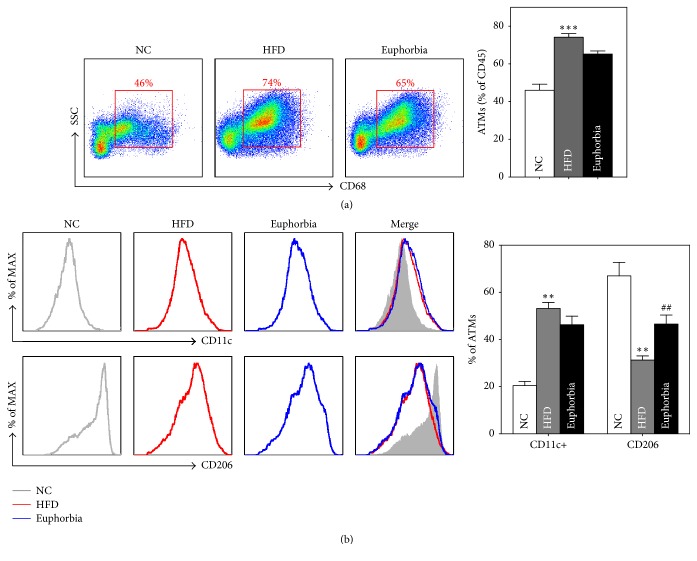
(a) ATM infiltration rate; (b) CD206- and CD11c-positive ATMs. NC: NC group; HFD: HFD group;* Euphorbia*:* Euphorbia* group. ^*∗∗*^*p* < 0.01; ^*∗∗∗*^*p* < 0.001 versus NC group. ^##^*p* < 0.01 versus HFD group.

**Figure 3:**
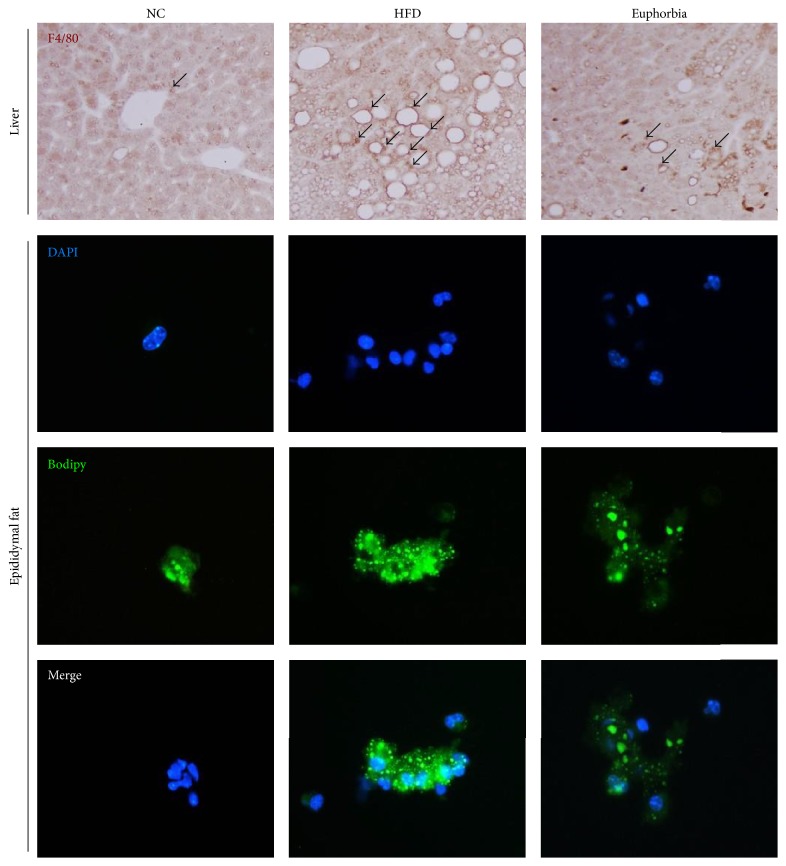
Immunohistochemical staining (F4/80) of liver and immunofluorescence staining of stromal vascular cells. Arrow indicates F4/80 positive Kupffer cells. NC: NC group; HFD: HFD group;* Euphorbia*:* Euphorbia* group.

The F4/80 positive Kupffer cells and fat deposits were markedly increased in HFD (F4/80-stained surface of 4.61 ± 0.64% in HFD versus 0.89 ± 0.18% in NC, *p* < 0.01), and the number of Kupffer cells and lipid accumulation were decreased in* Euphorbia* group (2.20 ± 0.22% in* Euphorbia* versus HFD, *p* < 0.05) (Figure 3). The RT-PCR analysis confirmed the markedly reduced the expression of CD68 mRNA in liver (Figure 1(c)). CD68 positive ATMs infiltration rate was significantly higher in the HFD group compared to the NC group (*p* < 0.001), and* Euphorbia* showed significant decrease in ATMs percentage (*p* < 0.05) (Figure 2(a)). The percentage of CD11c+ ATMs was significantly higher in the HFD group compared to the NC group (*p* < 0.05) and lower in the* Euphorbia* group compared to the HFD group, but the difference was not significant. In contrast, the CD206+ ATMs percentage was significantly lower in the HFD group than the NC group (*p* < 0.01) and significantly higher in the* Euphorbia* group than the HFD group (*p* < 0.01) (Figure 2(b)).

### 3.6. Immunofluorescence Staining of SVCs

Compared to the NC group, the HFD group showed an increase in the number of stained macrophages and lipid accumulation. For the* Euphorbia* group, the number of macrophages and lipid accumulation were decreased (Figure 3).

## 4. Discussion

In this study, we evaluated* Euphorbia* as a potential therapy for obesity and insulin resistance. We were able to obtain data on efficacy and safety of* Euphorbia* from human studies, since* Euphorbia* has already been approved for use as herbal medicine in the Republic of Korea when used by a professional physician [18]. Additionally, we conducted an* in vivo* study to estimate the efficacy, safety, and mechanism of* Euphorbia*, especially with respect to the anti-inflammatory effect.

In a study of 14 obese patients, we found that a 1-day prescription of 6 g* Euphorbia* caused significant decrease in BW and BMI. Because* Euphorbia* is a cathartic herbal medicine, the short-term effect of* Euphorbia* might mostly result from dehydration. However, in our human study, the weight loss effects persisted for one month, which allowed enough time to recover from dehydration. This result implies that the effect of* Euphorbia* depends on more than simple dehydration induced by diarrhea.

It was reported that administration of* Euphorbia* with herbal decoction to obese rats induced weight loss [17]. In agreement, administration of* Euphorbia* in the present study induced significant decrease in BW and epididymal fat weight, which is equivalent to visceral fat in humans. A decrease in epididymal fat weight after a five-week administration of* Euphorbia* in mice demonstrated the antiobesity effect of* Euphorbia*. In a previous study [16] of ethanol extracts of* Euphorbia*, a new tetracyclic triterpenoid of* Euphorbia* and five known triterpenoids with euphane skeletons were shown to be potentially useful in the clinical treatment of diabetes. In this study,* Euphorbia* showed antihyperglycemic effects on FBG and OGTT of obese mice. In addition, changes in FI and HOMA-IR indicate that* Euphorbia* improves IR.

It has been shown that high-fat diet increases Kupffer cells number and induces their proinflammatory status, and cytokine and chemokine production by activated Kupffer cells is involved in the pathogenesis of liver damage [19]. Moreover, visceral adipose tissue is an endocrine organ that releases adipokines and cytokines, which have a role in fat metabolism and insulin sensitivity [20]. Particularly, macrophage accumulation with increased cytokines was observed in adipose tissue, which implies the chronic inflammatory features of obesity [21, 22]. In this study,* Euphorbia* administration was related to a significant decrease in gene expression of cytokines TNF-*α*, IFN-*γ*, and IL-6. TNF-*α* is known to reduce insulin sensitivity by impairing insulin signal transduction [23]. Also, an increase in the expression of IFN-*γ* and IL-6 was also associated with insulin resistance [24, 25].

The rate of Kupffer cells in liver and ATM infiltration into adipose tissue was also decreased in the* Euphorbia*-treated group. Particularly, the percentage of CD206+ ATMs, which indicates the anti-inflammatory activity of ATMs in adipose tissue, was significantly increased in the* Euphorbia*-treated group in comparison to the HFD group [26]. This result suggests that improvement of hyperglycemia and IR in the* Euphorbia* group is correlated with the anti-inflammatory effects of* Euphorbia* on liver and visceral adipose tissue.

In obesity, adipocyte-hypertrophy leads to hypoxia, endoplasmic reticulum stress, and lipotoxicity, which result in increased macrophage infiltration accompanied by elevated concentrations of inflammatory markers. The accumulation of macrophages in adipose tissue leads to a specific feature called crown-like structure, which is the cluster of macrophages around dead adipocytes [27]. We obtained SVCs from the epididymal fat pad and analyzed them using immunofluorescence microscopy. The SVCs from the* Euphorbia* group showed a decreased number of macrophages with a smaller amount of lipid accumulation compared to those in the HFD group.

Taken together, the degree of lipid accumulation in liver and visceral fat, Kupffer cells, ATM infiltration rate, which produces proinflammatory cytokines in liver and adipose tissue, and gene expression of cytokines are important for evaluating antiobesity, anti-inflammatory, and anti-IR effects. In this study, the BW decrease caused by* Euphorbia* treatment persisted for 1 month in humans, and the* in vivo* studies showed epididymal fat weight decrease, decline in Kupffer cells and ATM percentage, and decreased expression of TNF-*α*, IFN-*γ*, and IL-6 genes. These results indicate that* Euphorbia* has an antiobesity effect, which consequently induces an anti-inflammatory effect that improves IR.

## 5. Conclusion

In conclusion, our study showed that* Euphorbia* improves body weight and insulin resistance in human and* in vivo* study. A possible mechanism to explain this result is the reduction in the inflammatory reaction due to decreased Kupffer cells and adipose tissue macrophage infiltration. The cathartic effects of* Euphorbia* necessitate further research to assess issues with diarrhea that might affect long-term use, even though the results suggest that* Euphorbia* can be used to effectively improve obesity-related IR.

## Figures and Tables

**Table 1:** Comparison of outcome measures in an obese human study.

	Before	After	*p* value
BW (kg)	65.37 ± 3.53	64.10 ± 3.49	0.02
BMI	24.93 ± 0.65	24.28 ± 0.58	0.047
Waist-hip ratio	0.90 ± 0.01	0.90 ± 0.01	0.79
Percent body fat (%)	29.71 ± 1.44	29.56 ± 1.39	0.58
BUN (mg/dl)	15.28 ± 1.32	14.52 ± 1.14	0.44
Creatinine (mg/dl)	0.72 ± 0.39	0.72 ± 0.42	0.75
eGFR	97.38 ± 6.71	93.58 ± 4.94	0.36
AST (U/L)	32.42 ± 6.96	27.04 ± 2.53	0.51
ALT (U/L)	35.25 ± 13.56	25.96 ± 5.40	0.28
GGT (U/L)	44.91 ± 13.53	43.04 ± 13.49	0.77
Protein (g/dl)	6.98 ± 0.53	6.79 ± 0.55	0.21
Albumin (g/dl)	3.99 ± 0.32	3.98 ± 0.48	0.97
Fasting glucose (mg/dl)	94.5 ± 5.6	88.3 ± 9.99	0.12
HbA1c (%)	6.11 ± 0.58	5.97 ± 0.56	0.28

“Before” and “after” indicate before *Euphorbia* administration and after *Euphorbia* administration, respectively. Data are presented as mean ± SE. For *p* value, the results are from paired *t*-test to examine within-group effects. BW: body weight; BMI: body mass index; BUN: blood urea nitrogen; eGFR: CockCroft-Gault estimated glomerular filtration rate; AST: aspartic aminotransferase; ALT: alanine aminotransferase; GGT: gamma glutamyltranspeptidase.
